# Evaluation of tolerability and safety of transcranial electrical stimulation with gel particle electrodes in healthy subjects

**DOI:** 10.3389/fpsyt.2024.1441533

**Published:** 2024-11-13

**Authors:** Chuangchuang Chang, Yi Piao, Mingsong Zhang, Yan Liu, Minglei Du, Miao Yang, Tianyuan Mei, Chengkai Wu, Yan Wang, Xueli Chen, Ginger Qinghong Zeng, Xiaochu Zhang

**Affiliations:** ^1^ Application Technology Center of Physical Therapy to Brain Disorders, Institute of Advanced Technology, University of Science & Technology of China, Hefei, China; ^2^ School of Biomedical Engineering, Division of Life Sciences and Medicine, University of Science and Technology of China, Hefei, Anhui, China; ^3^ Division of Life Science and Medicine, University of Science & Technology of China, Hefei, China; ^4^ Department of Radiology, the First Affiliated Hospital of USTC, Hefei National Research Center for Physical Sciences at the Microscale and School of Life Science, Division of Life Science and Medicine, University of Science & Technology of China (USTC), Hefei, China; ^5^ Key Laboratory of Brain-Machine Intelligence for Information Behavior (Ministry of Education and Shanghai), School of Business and Management, Shanghai International Studies University, Shanghai, China; ^6^ Institute of Health and Medicine, Hefei Comprehensive Science Center, Hefei, China; ^7^ Business School, Guizhou Education University, Guiyang, China

**Keywords:** gel, tDCS, tACS, stimulation type, stimulation duration, tolerability, safety

## Abstract

**Background:**

With the advancement of transcranial electrical stimulation (tES) technology, an increasing number of stimulation devices and treatment protocols have emerged. However, safety and tolerability remain critical concerns before new strategies can be implemented. Particularly, the use of gel particle electrodes brings new challenges to the safety and tolerability of tES, which hinders its widespread adoption and further research.

**Objective:**

Our study utilized a specially designed and validated transcranial electrical stimulation stimulator along with preconfigured gel particle electrodes placed at F3 and F4 in the prefrontal lobes. We aimed to assess the tolerance and safety of these electrodes in healthy subjects by administering different durations and types of tES.

**Methods:**

Each participant underwent ten sessions of either transcranial direct current stimulation (tDCS) or transcranial alternating current stimulation (tACS), with session durations varying. In the experiment, we collected various measurement data from participants, including self-report questionnaire data and behavioral keystroke data. Tolerability was evaluated through adverse events (AEs), the relationship of adverse events with tES (AEs-rela), the Self-Rating Anxiety Scale (SAS), and the Visual Analog Mood Scale-Revised (VAMS-R). Safety was assessed using the Visual Analog Scale (VAS), the Skin Sensation Rating (SSR), Montreal Cognitive Assessment (MoCA), and Stroop task. These data were analyzed to determine the impact of different parameters on the tolerability and safety of tES.

**Results:**

There were no significant changes in the results of the MoCA and SAS scales before and after the experiment. However, significant differences were observed in VAS, SSR, AEs, and AEs-rela between tDCS and tACS. Additionally, fatigue increased, and energy levels decreased on VAMS-R with longer durations. No significant differences were found in other neuropsychological tests.

**Conclusion:**

Our study revealed significant differences in tolerability and safety between tDCS and tACS, underscoring the importance of considering the stimulation type when evaluating these factors. Although tolerance and safety did not vary significantly across different stimulation durations in this study, future research may benefit from exploring shorter durations to further assess tolerability and safety efficiently.

## 1 Introduction

The transcranial electrical stimulation (tES) is a non-invasive neurostimulation technique, which typically includes transcranial direct current stimulation (tDCS) and transcranial alternating current stimulation (tACS) (1–3). It has garnered rapid attention and promotion owing to its ease of use, non-invasiveness, adjustability, and broad range of applications (4–6). Over the past five years, more than 5000 articles have been published on this subject (7). In most clinical scenarios, cortical excitability is modulated by placing one or more pairs of electrodes (8) on the surface of the human scalp, with an applied current intensity of 1-2 mA (9, 10). The tES is generally regarded as non-invasive and well-tolerated by subjects with favorable safety profiles (11–13). However, the weak current produces a low-strength electric field (14), necessitating long-term repetition and accumulation to achieve desired effects. Enhancing the intensity of tES shows the potential to yield improved effectiveness (15–19). Nevertheless, the heightened skin sensation and potential side effects impede further enhancement of the stimulation intensity (15, 16, 20–22).

Numerous types of electrodes exist today that can be utilized for tES, including the commonly used comb dry electrode, sponge saltwater electrode, and gold standard conductive paste electrode. Dry electrodes are easy to use, but have problems with high contact impedance and lack of comfort (23, 24). In contrast, wet electrodes have low contact impedance, but their operation process is time-consuming, and the evaporation of water after long-term use will cause the impedance to increase (25, 26). Moreover, the process of cleaning hair and electrode caps adds to the complexity. Gel electrodes combine the low contact impedance characteristics of wet electrodes with better moisture retention, efficient conductivity and stability (24, 27–29). Unlike wet electrodes, gel electrodes do not leave residues, require minimal preparation time, and do not adhere to the skin (30, 31). Additionally, they maintain relatively stable impedance over extended periods, making them suitable for prolonged brain stimulation experiments. Recently, gel electrodes, as a semi-dry alternative that balances portability and high performance, have seen widespread application in brain stimulation. It achieves a good balance between ease of use and conductivity performance (22, 27, 30). Regardless of the stimulation conditions, ensuring robust safety and tolerability in participants is essential for widespread acceptance (11, 32–34).

The application of tES using gel electrodes, selecting appropriate stimulation parameters is crucial, including stimulation duration, stimulation type, current intensity, and stimulation frequency, among others. Stimulation duration is an important factor that influences tolerability and safety. Especially for potential long-term applications of tES devices, such as chronic therapy or daily interventions. It directly impacts skin responses, cognitive load, and long-term effects on the body (20). Several studies stimulated for more extended periods than 20 minutes without reporting side effects (35). In the tES safety guidelines from Antal et al., tES has been established as safe for up to 60 min duration per day (36). Nevertheless, Alonzo et al. proposed that the repeated tES sessions may result in longer stimulation duration (37), ultimately leading to increased cumulative energy transmission to the brain. This could potentially heighten the risk of heat injury, electrolysis, or fatigue-related discomfort. Conversely, a short duration of stimulation may not be sufficient to induce significant neuromodulation effects (38). Therefore, uncertainty remains regarding the relationship between stimulation duration and both safety and tolerability, especially within the framework of different tES paradigms and individual variances. With the increasing emergence of tES devices, it is essential to study the tolerability and safety of these new devices and treatment modalities before their widespread adoption. However, when designing experiments, multiple variables must often be considered, such as the treatment environment, target population, application methods, and stimulation parameters. The diversity of these conditions, combined with the duration of stimulation, collectively determines the overall duration of the experiment. In this context, stimulation duration becomes a critical variable in research, as it not only dictates the structure and progression of the experiment but also directly impacts the deployment of devices and protocols (39).

Although stimulation duration is a key focus of research, the influence of stimulation type is equally important. In the field of tES, the most commonly used stimulation types are tDCS and tACS (1–3). tDCS can administer a consistent, unidirectional flow of current to influence nerve membrane polarization, regulate the resting membrane potential, and modulate synaptic transmission (40–42), thus altering brain excitability. In contrast, tACS delivers an oscillating current that alternates between positive and negative polarities periodically, potentially synchronizing or interacting with endogenous brain rhythms (43–45). Due to variations in the mechanisms of action between these two types of tES, the physiological responses, side effects, and subjective experiences elicited by each technique may differ, even when employing the same current intensity (46, 47). Previous studies have examined the tolerability and safety of tDCS or tACS using various devices and treatment protocols (11, 16, 20, 47). However, comparative research on the tolerability and safety of these two types of tES using gel-based electrodes remains limited, and no consensus has been reached (48, 49).

In this context, the present study aims to investigate the effects of stimulation duration and type on the tolerability and safety of tES using gel-based electrodes. This research will provide valuable reference points for selecting appropriate stimulation parameters in the design and clinical application of tES devices, laying the foundation for tailoring stimulation protocols to different clinical indications.

## 2 Materials and methods

In this study, tES was performed using preconfigured gel particle electrodes to gather experimental measurements, including self-report questionnaire data and in-experiment behavioral keystroke data from participants. These measurements included adverse events (AEs) and the relationship of adverse events with tES (AEs-rela) in terms of tolerability, the Visual Analog Scale (VAS) and the Skin Sensation Rating (SSR) ratings in terms of safety, mental health evaluation and behavioral experimental outcomes from the Montreal Cognitive Assessment (MoCA), the Self-Rating Anxiety Scale (SAS), the Visual Analog Mood Scale-Revised (VAMS-R), and Stroop task performance data.

### 2.1 Participants

In the study, 17 healthy subjects were recruited, comprising 6 females and 11 males. One female was excluded due to a history of head nerve pain, and one male was excluded due to data collection errors related to experimental parameters. Ultimately, the remaining 15 healthy subjects (5 females and 10 males, mean age ± SD: 24.00 ± 1.36 years, average education duration: 17.53 ± 1.19 years.) successfully completed the entire experiment.

All participants with normal or corrected-to-normal vision provided their informed consent forms and basic information forms, and specified the scales and behavioral tasks to be completed during the experiment. Prior to the experiment, no participants reported a history of craniotomy or head injury, personal or family history of neurological or psychiatric disorders, metal implants or implanted electronic devices, skin sensitivities, or drug use. For safety reasons, individuals who are pregnant or may become pregnant were excluded from participation. Informed consent was obtained before engaging in the research. This study was approved by the Human Ethics Committee of the University of Science and Technology of China (IRB No. 2022KY275).

### 2.2 Experimental procedure

Participants voluntarily participated in a total of 10 stimulation sessions, comprising five sessions of tDCS and five sessions of tACS, each stimulus type with varying durations (2 minutes, 5 minutes, 7 minutes, 10 minutes, and 20 minutes). In our study, the interval between each stimulation session is 5 minutes. No washout period was included. Therefore, we implemented a Latin square design to balance the sequence of experimental conditions. This design ensures that the effects of different conditions are evenly distributed across participants, effectively minimizing potential carryover or order effects on the results. The experiment adhered to a single-blind multi-condition experimental approach.

**Figure 1:**
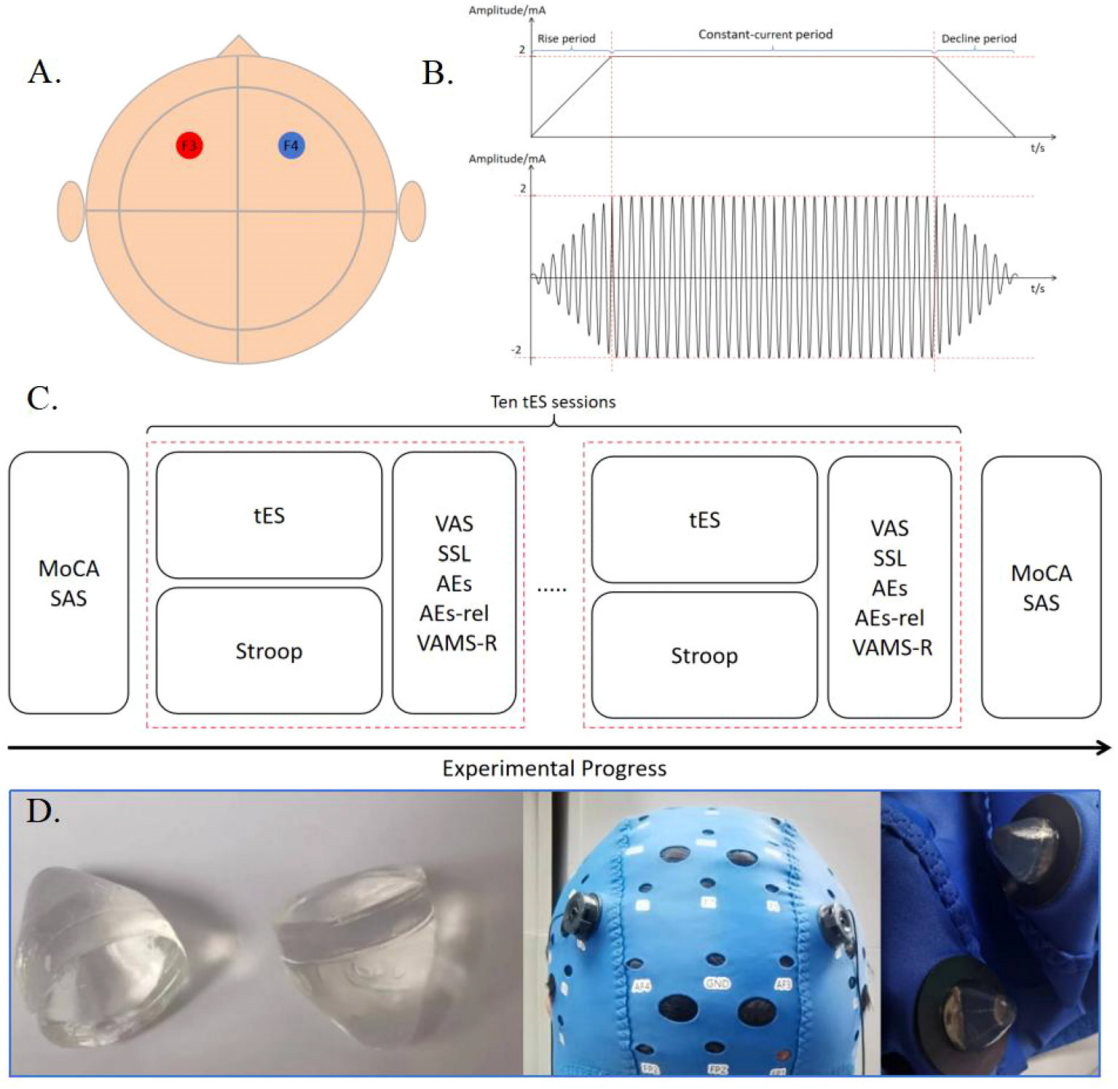
**(A)** Locations of the stimulation electrodes. The positive and negative electrodes of a pair are placed in F3 and F4, respectively. **(B)** Schematic diagram of tDCS and tACS stimulation with a current intensity of 2mA. **(C)** Experimental Procedure. **(D)** Preset gel particle electrode used in this experiment.

Participants were instructed to complete neuropsychological scales such as MoCA and SAS prior to the formal stimulation. During the stimulation process, participants engaged in a behavioral keystroke Stroop task and stopped the Stroop task at the end of the stimulation. The VAS, SSR, AEs, AEs-rela, and VAMS-R scales were completed immediately after each stimulation session. After the completion of all the stimulations, each subject underwent neuropsychological scale assessments such as MoCA and SAS, as illustrated in **Figure 1**.

### 2.3 Transcranial electrical stimulation

The selected electrode is a preconfigured gel particle electrode, which is connected to the output port of the stimulator using a silver/silver chloride powder sintered electrode and a gel-fixed shell, with a diameter of 10 mm. It is secured on the scalp using a professional EEG electrode cap, ensuring that the impedance value during electrical stimulation remains below 15 kΩ. tDCS was set to 2 mA, and tACS was set to 2 mA at 10 Hz. The current linearly increased to 2 mA for 30 seconds at the beginning of the stimulation and then decreased to 0 mA for 30 seconds at the end of the stimulation. The intensity of stimulation at 2 mA was maintained throughout the duration of this study. Real-time monitoring of impedance changes during the stimulation process is conducted by analyzing impedance return values from software on the tablet. If the impedance levels become too high, the relay will be automatically disconnected to protect the subject. At the end of each stimulation session, participants were required to complete a self-reported preparation form before commencing the next stimulation program. Participants were seated in a comfortable chair, which they adjusted according to their preference, facing a screen displaying the Stroop task.

The transcranial electrical stimulation device utilized is a lithium battery-powered stimulator. The stimulator communicates with the software on the tablet computer via Bluetooth, and the software establishes specific parameters, which are then transmitted to the stimulator for execution. The stimulator has been isolated from the main power supply, and batteries are utilized to ensure the safety of the subjects. The performance of the stimulator has been previously verified in the literature (50). The F3 and F4 electrodes in the 10-20 EEG system were selected as stimulation sites in this experiment, which are located in the left and right prefrontal lobes, respectively. The reason of the selection of the stimulation site was that the function of the brain region underlying the F3 and F4 electrodes aligns with the cognitive abilities verified by the Stroop task (51, 52).

The participant is blinded to the stimulus conditions, and the control software for the stimulus parameters is installed on a tablet computer, ensuring that the participant does not have access to information regarding the stimulus parameters.

### 2.4 Neuropsychology tests

The MoCA Scale is a convenient and efficient cognitive screening tool that encompasses various cognitive domains, including attention and concentration, executive function, memory, language, visual-spatial skills, abstract thinking, calculation, and orientation. To minimize the impact of pre-test memory on subjects’ performance, an alternate version of the test was administered both before and after the experiment.

The SAS scale is a psychometric tool utilized for assessing anxiety state and severity. Participants respond to a series of self-reported questions regarding feelings of anxiety, encompassing sensations such as nervousness, worry, fear, and physical discomfort. Each item is rated on a Likert scale from 1 to 4 (1 = absent or rarely present, 2 = sometimes present, 3 = mostly present, 4 = almost always or always present). They then select a rating that best corresponds to their current situation. Subsequently, the scores for each item are totaled and calculated to derive an overall score. This score is used to determine the presence of anxiety disorders and evaluate the level of anxiety symptoms in individuals.

The VAMS-R scale were utilized to assess the emotional states of the participants. Emotional states, including changes in visual perception of sorrow, bewilderment, fear, happiness, fatigue, anger, strain, and full of energy, were gauged by indicating the intensity of feelings on a specified emotional continuum line. After measurement and quantification, each item was scored on a scale ranging from 0 to 100, with a high score indicating that emotion was strong and a low score indicating the opposite.

### 2.5 The Stroop task

The Stroop task, also known as the Stroop Effect experiment, is a classic psychological study initially proposed by John Ridley Stroop in 1935 (53). This experiment is primarily utilized to investigate human cognitive processes, particularly in the areas of attention, perception, reaction time, and executive function. In this study, the performance in the Stroop task was used to indicate whether subjects concentrate enough during the experiment (51, 54).

The Stroop task used in this study was compiled by PsychoPy 3.8.10. Instructions will be displayed when it was initiated. Please wait until the participant is ready and press the ‘space’ key to begin. A series of color words will appear on the screen. However, it is important to note that the task is not to read the meaning of the word, but rather to quickly and accurately report the color of the word by pressing the corresponding button. Specifically, ‘red’ corresponds to the key ‘R’, ‘green’ corresponds to the key ‘G’, ‘blue’ corresponds to the key ‘B’, and ‘yellow’ corresponds to the key ‘Y’.

The experimental procedure involved consistent and inconsistent colors. Participants were instructed to focus on the colors and ignore the words. Clear prompts were provided between the beginning of the experiment and each stage. If participants had any questions, they were encouraged to ask the interviewer. After becoming familiar with the task, participants were instructed to promptly and accurately identify and provide feedback on the word font color in the formal stimulus experiment. Following the conclusion of the experiment, data on both accuracy rate and reaction time were collected by the Stroop task stop program from participant responses. This study focuses specifically on two key results: percentage of correct responses and reaction time.

### 2.6 Sensation and adverse events

The pain score is measured by the VAS, which ranges from 0 to 10 points from left to right. A higher score indicates a greater level of pain. The relation between the score and pain intensity is as follows: 1-3 = mild pain; minimal impact on activities of daily living; 4-6 = moderate pain; moderate impact on the ability to live daily; 7-10 = severe pain; significant impact on the ability to live daily.

The SSR scale was used to indicate the tolerance effect of tES on the subject by providing evaluation options of ‘no sensation,’ ‘slight,’ ‘moderate,’ ‘severe,’ and ‘extreme,’ corresponding to discrete values of 1, 2, 3, 4, and 5, respectively. Subjects are allowed to stop the stimulation process at any time if they are unable to tolerate it.

AEs were evaluated for symptoms such as headache, neck pain, scalp pain, scalp pressure, tingling, burning sensation, itching sensation, sleeping issues, trouble concentrating, dizziness, nausea, and vibration intensity on a scale of 1 to 4 (1 = absent, 2 = mild, 3 = moderate, 4 = severe). Simultaneously, participants rated the relationship between adverse events and tES (AEs-relation) on a scale of 1 to 5 (1 = none, 2 = remote, 3 = possible, 4 = probable, 5 = definite).

### 2.7 Statistical analysis

All analyses were conducted using OriginPro 2021 software (OriginLab Corp, Northampton, MA, USA) and IBM SPSS Statistics 26.0 (IBM Corp, Armonk, NY, USA). Based on the normality and homogeneity of variance of the data in each condition, the paired-sample Wilcoxon signed-rank test with non-parametric analysis was utilized for the comparison between tDCS and tACS. Friedman analysis of variance (ANOVA) was performed for the comparison between five stimulation durations.

## 3 Results

By applying tES to F3 and F4 on the scalp surface of healthy subjects using preset gel particle electrodes, we investigated the safety and tolerability of stimulation types (tDCS and tACS), as well as stimulation durations (2 minutes, 5 minutes, 7 minutes, 10 minutes, and 20 minutes). In the study, MoCA, SSA, VAS, and SSR, AEs, AEs-rela, VAMS-R and Stroop analysis based on participants’ rating and response were analyzed.

**Figure 2:**
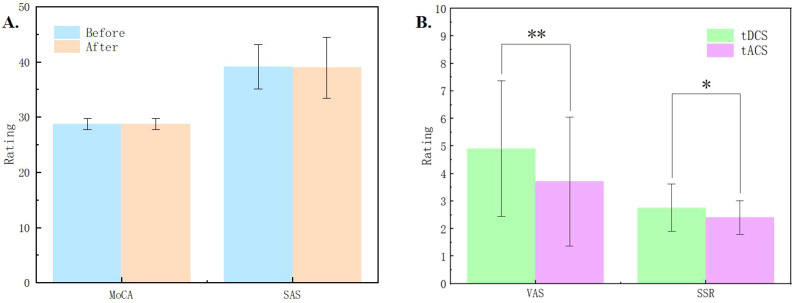
**(A)** Results of the Montreal Cognitive Assessment (MoCA) and Self-Rating Anxiety Scale (SAS) before and after the experiment. **(B)** Comparisons of the pain measured by Visual Analog Scale (VAS) and skin sensation rating (SSR) between tDCS and tACS. * indicates p < 0.05, ** indicates p < 0.01.

The results of MoCA and SAS scales showed no significant changes in cognitive function and anxiety levels before and after the stimulation sessions (see **Figure 2A**), suggesting that the subjects remained in a cognitively normal and non-anxious state throughout the study. The accuracy in the Stroop task under different conditions was larger than 85%, with an averaged accuracy was larger than 97%, indicating that subjects were quite engaged in the experiment, minimizing potential discrepancies in self-reported outcomes due to distractions (15).

### 3.1 Stimulation duration

For the VAS, and SSR, AEs, AEs-rela, VAMS-R and Stroop, no significant differences were found between stimulation durations (p > 0.05), except for the fatigue item and the full of energy item of the VAMS-R (see **Supplementary Table S1**). Specifically, with the increase in the duration of stimulation, fatigue of subjects significantly increased (χ2 = 13.667, p = 0.008), while energy significantly decreased (χ2 = 9.906, p = 0.042).

### 3.2 Stimulation type

In the experiment, we collected VAS pain ratings and skin sensation ratings of the subjects in each session. The average VAS rating was below 5 points, and the skin sensation rating was also below 3 points. The results indicated that tDCS was significantly higher than tACS in terms of both VAS (*Z* = 2.867, p = 0.001) and SSR (*Z* = 2.048, p = 0.033) ratings, and subjects felt more painful and less tolerable under the stimulation of tDCS (see **Figure 2B**).

**Figure 3:**
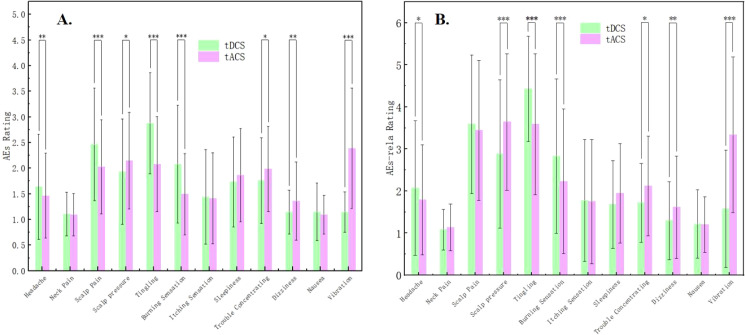
**(A)** Comparisons of Adverse events (AEs) between tDCS and tACS. **(B)** Comparisons of Adverse events relation (AEs-rela) of tES between tDCS and tACS. * indicates p < 0.05, ** indicates p < 0.01, *** indicates p < 0.001.

For the AEs, significant differences were found between tDCS and tACS for headache, scalp pain, scalp pressure, tingling, burning sensation, trouble concentrating, dizziness, and vibration (*Z* = -5.976 to 5.526, p = <0.001 to 0.029). Specifically, headache, scalp pain, tingling, and burning sensation were more intense under tDCS than those under tACS. On the other hand, scalp pressure, trouble concentrating, dizziness, and vibration were more intense under tACS than under tDCS (see **Supplementary Table S2**; **Figure 3A**). Detailed data revealed that adverse events with a rating higher than half of the total value included scalp pain, tingling, and burning sensation under tDCS, as well as scalp pain, scalp pressure, tingling, and vibration under tACS.

For the AEs-relation, consistent with those for AEs, significant differences between tDCS and tACS were found regarding headache, scalp pressure, tingling, burning sensation, trouble concentrating, dizziness, and vibration (*Z* = -5.569 to 4.495, p = <0.001 to 0.013), indicating that subjects believed that these adverse effects were more likely to be related to the stimulation of tDCS or tACS (**Supplementary Table S2**; **Figure 3B**). Specifically, the belief of the relation between AEs and tES was stronger for tDCS than tACS regarding headache, tingling, and burning sensation while weaker regarding scalp pressure, trouble concentrating, dizziness, and vibration.

**Figure 4:**
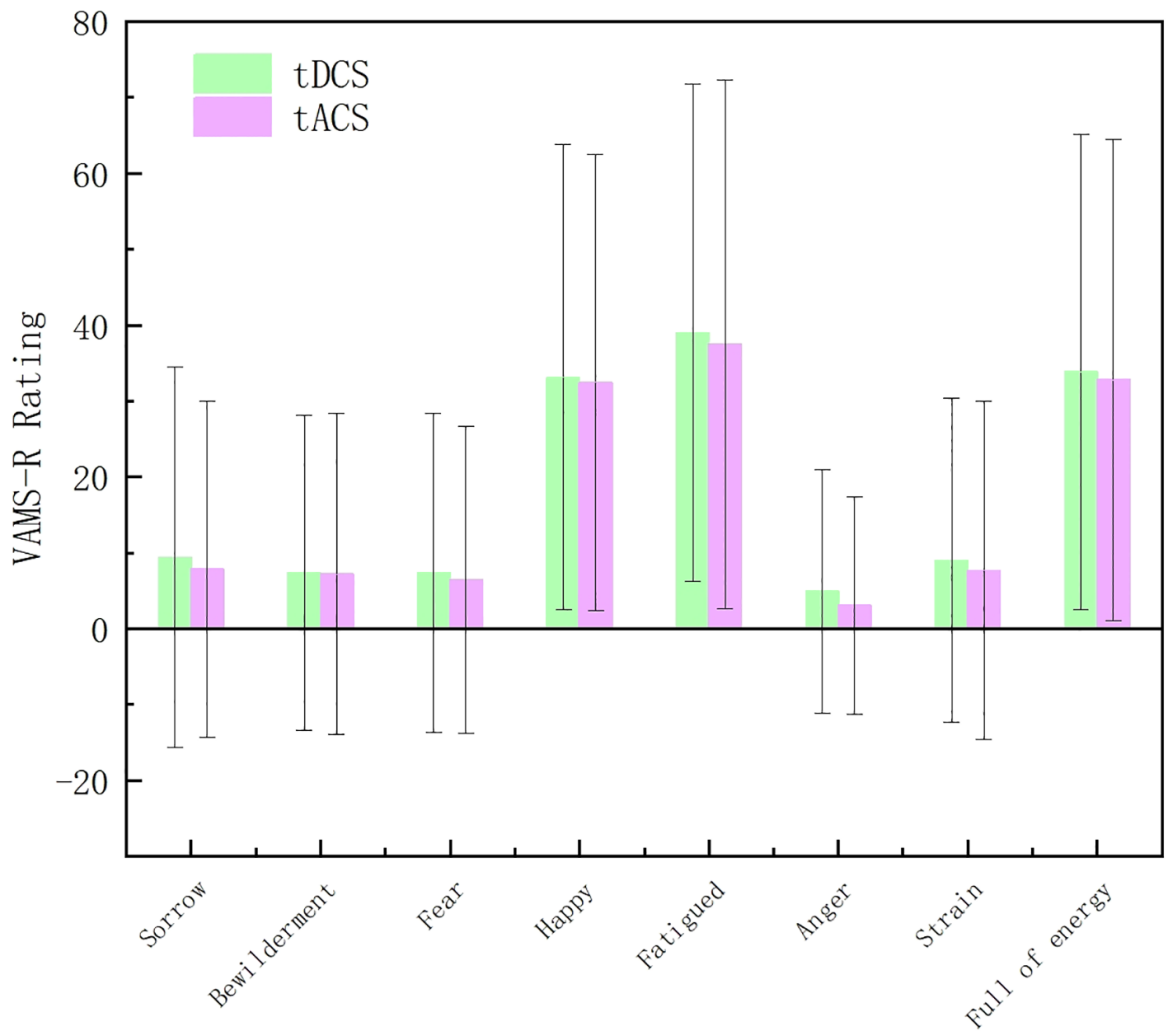
Comparisons of items in Visual Analogue Mood Scales-Revised (VAMS-R) between tDCS and tACS.

**Figure 5:**
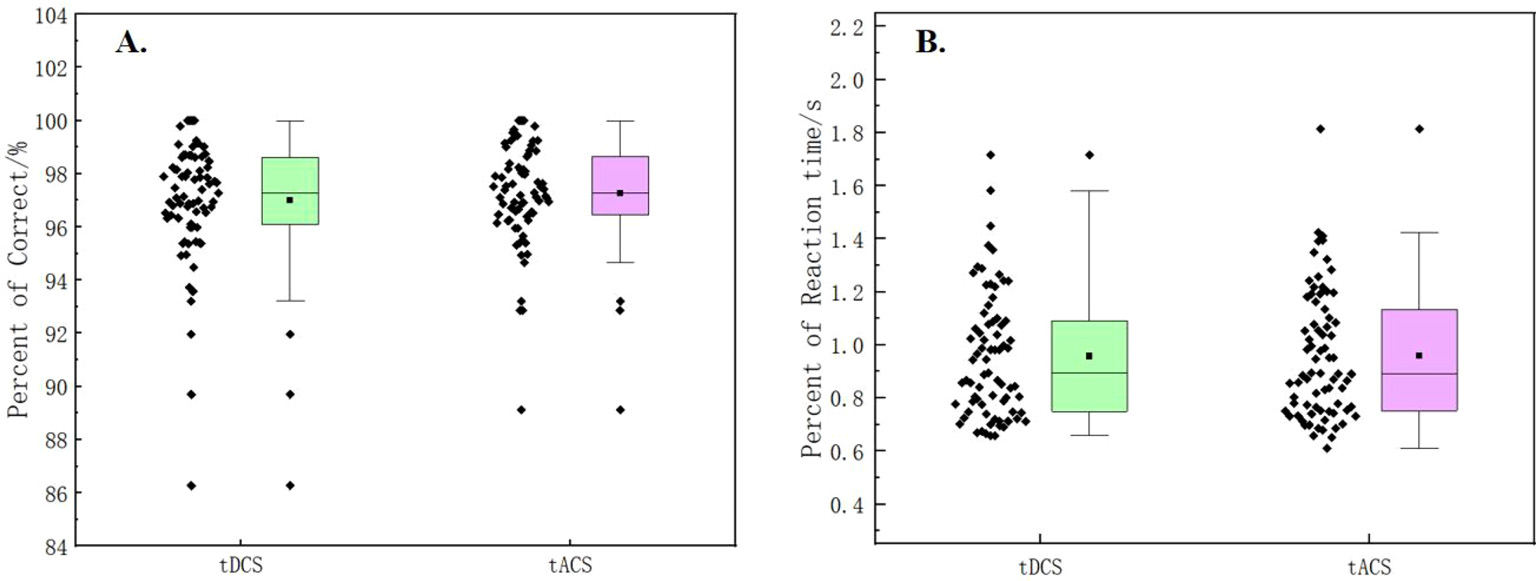
**(A)** Comparisosn of percent of correct in the Stroop task between tDCS and tACS. **(B)** Comparisons of reaction time in the Stroop task between tDCS and tACS.

In this experiment, the subjects were evaluated for their emotional responses after each stimulation session. The result shown in **Figure 4** indicated that there was no significant difference in emotional response between tDCS and tACS. As illustrated in **Figure 5**, the Stroop behavioral keystroke experiment showed no significant variance across different stimulus types.

## 4 Discussion

With the advancement of technology, gel particle electrodes have gradually emerged in our field of vision. Due to their portability, comfort, hygiene, and ease of operation, they have become an important option for brain-computer interface electrodes. In this study, we examine the tolerability and safety with 2mA current of different stimulation durations and types using preset gel particle electrodes. There were no significant differences between different durations but significant differences between stimulation types, stressing on the attention to the stimulation type rather than stimulation durations when accessing the tolerability and safety.

For the influence of stimulation durations, there were no significant differences in tolerance and safety except for emotions like fatigued and energetic, indicating that tolerability and safety in this study remained stable regardless of the duration of stimulation. Since it was found that participants experienced a significant increase in fatigue and a significant decrease in feelings of being full of energy, the prolonged stimulation may have a negative impact on the emotional state of the participants, particularly in terms of fatigue. The findings suggests that subjects can adapt to tES stimulation with increasing time, which has significant implications for the design of long-term use of the device or repeated stimulation protocols. It also provides a reference for selecting experimental durations for future experimental methods and devices aiming to test tolerability and safety.

By analyzing the scores of the MoCA and SAS scales, it is evident that this experiment does not induce changes in mild cognitive impairment or anxiety state among the subjects. This indicates that both the experimental design and tES itself have no obvious harmful psychological effects on the participants. Furthermore, statistical analysis of VAS, SSR, and AEs suggests that tDCS brought about more changes in pain perception in subjects, while the vibration and other sensations brought about by tACS may be more influenced by the characteristics of the AC current waveform. However, there was no discernible difference between the two types of stimuli in terms of emotional responses and Stroop performance. Generally, the tolerability and safety of tDCS were found to be inferior to that of tACS in this study conditions.

The current study has some limitations. The study only targeted basic tES and did not investigate the potential effects of more stimulating parameters such as electrode position, duty cycle, and waveform on tolerance and safety. Limited by the total stimulus duration, this study did not include longer stimulus durations of more than 20 minutes per session. The sample size was small, and the subjects were mostly from universities, so future studies should consider selecting a larger and more diverse sample to examine differences across various subjects. Furthermore, the study was focused on the behavioral data, lacking biomarkers to further explore neurophysiological mechanisms.

## 5 Conclusion

The study demonstrates significant differences in tolerability and safety between tDCS and tACS when using preset gel particle electrodes, suggesting that the choice of stimulation type should be tailored to individual differences. There were no significant differences in tolerance and safety effects for different stimulation durations. It is recommended to select shorter durations when validating other transcranial electrical stimulation devices and treatment approaches to efficiently explore tolerability and safety.

## Data Availability

The original contributions presented in the study are included in the article/**Supplementary Material**. Further inquiries can be directed to the corresponding authors.

## References

[1] Mills KR Murray NMF Hess CW. Magnetic and electrical transcranial brain stimulation: physiological mechanisms and clinical applications. Neurosurgery. (1987) 20:164–8. doi: 10.1097/00006123-198701000-00033 3543723

[2] Woods AJ Antal A Bikson M Boggio PS Brunoni AR Celnik P. A technical guide to tDCS, and related non-invasive brain stimulation tools. Clin Neurophysiol. (2016) 127:1031e48. doi: 10.1016/j.clinph.2015.11.012 26652115 PMC4747791

[3] Antal A Boros K Poreisz C Chaieb L Terney D Paulus W. Comparatively weak after-effects of transcranial alternating current stimulation (tACS) on cortical excitability in humans. Brain Stimulation. (2008) 1:97–105. doi: 10.1016/j.brs.2007.10.001 20633376

[4] Kaelin-Lang A Cohen LG. Enhancing the quality of studies using transcranial magnetic and electrical stimulation with a new computer-controlled system. J Neurosci Methods. (2000) 102:81–9. doi: 10.1016/S0165-0270(00)00284-3 11000414

[5] Been G Ngo TT Miller SM Fitzgerald PB. The use of tDCS and cvs as methods of non-invasive brain stimulation. Brain Res Rev. (2007) 56:346–61. doi: 10.1016/j.brainresrev.2007.08.001 17900703

[6] Santarnecchi E Muller T Rossi S Sarkar A Polizzotto NR Rossi A. Individual differences and specificity of prefrontal gamma frequency-tACS on fluid intelligence capabilities. Cortex. (2016) 75:33–43. doi: 10.1016/j.cortex.2015.11.003 26707084

[7] Wischnewski M Alekseichuk I Opitz A. Neurocognitive, physiological, and biophysical effects of transcranial alternating current stimulation. Trends Cogn Sci. (2023) 27:189–205. doi: 10.1016/j.tics.2022.11.013 36543610 PMC9852081

[8] Soleimani G Kuplicki R Camchong J Opitz A Paulus MP Lim KO. Are we really targeting and stimulating DLPFC by placing transcranial electrical stimulation (tES) electrodes over F3/F4? Hum Brain Mapp. (2023) 44:6275–87. doi: 10.1002/hbm.26492 PMC10619406 37750607

[9] Wallace D Cooper NR Paulmann S Fitzgerald PB Russo R. Perceived comfort and blinding efficacy in randomised sham-controlled transcranial direct current stimulation (tDCS) trials at 2mA in young and older healthy adults. PloS One. (2016) 11:e0149703. doi: 10.1371/journal.pone.0149703 26900961 PMC4763202

[10] Kasten FH Duecker K Maack MC Meiser A Herrmann CS. Integrating electric field modeling and neuroimaging to explain inter-individual variability of tACS effects. Nat Commun. (2019) 10:5427. doi: 10.1038/s41467-019-13417-6 31780668 PMC6882891

[11] Paneri B Adair D Thomas C Khadka N Patel V Tyler WJ. Tolerability of repeated application of transcranial electrical stimulation with limited outputs to healthy subjects. Brain Stimulation. (2016) 9:740–54. doi: 10.1016/j.brs.2016.05.008 PMC5786157 27372844

[12] Gbadeyan O Steinhauser M Mcmahon K Meinzer M. Safety, tolerability, blinding efficacy and behavioural effects of a novel mri-compatible, high-definition tDCS set-up. Brain Stimulation. (2016) 9:547–54. doi: 10.1016/j.brs.2016.03.018 27108392

[13] Bland NS Sale MV. Current challenges: the ups and downs of tACS. Exp Brain Res. (2019) 237:3071–88. doi: 10.1007/s00221-019-05666-0 31620829

[14] Liu AL Vöröslakos M Kronberg G Henin S Krause MR Huang Y. Immediate neurophysiological effects of transcranial electrical stimulation. Nat Commun. (2018) 9:5092. doi: 10.1038/s41467-018-07233-7 30504921 PMC6269428

[15] Khadka N Borges H Paneri B Kaufman T Nassis E Zannou AL. Adaptive current tDCS up to 4mA. Brain Stimulation. (2020) 13:69–79. doi: 10.1016/j.brs.2019.07.027 31427272 PMC6888879

[16] Nitsche MA Bikson M. Extending the parameter range for tDCS: safety and tolerability of 4 ma stimulation. Brain Stimulation. (2017) 10:541–2. doi: 10.1016/j.brs.2017.03.002 PMC5972544 28456325

[17] Monte-Silva K Kuo M-F Hessenthaler S Fresnoza S Liebetanz D Paulus W. Induction of late LTP-like plasticity in the human motor cortex by repeated non-invasive brain stimulation. Brain Stimulation. (2013) 6:424–32. doi: 10.1016/j.brs.2012.04.011 22695026

[18] Batsikadze G Moliadze V Paulus W Kuo MF Nitsche M. Partially non-linear stimulation intensity-dependent effects of direct current stimulation on motor cortex excitability in humans. J Physiol-London. (2013) 591:1987–2000. doi: 10.1113/jphysiol.2012.249730 23339180 PMC3624864

[19] Bikson M Inoue M Akiyama H Deans JK Fox JE Miyakawa H. Effects of uniform extracellular DC electric fields on excitability in rat hippocampal slices in vitro. J Physiol-London. (2004) 557:175–90. doi: 10.1113/jphysiol.2003.055772 PMC1665051 14978199

[20] Hsu G Farahani F Parra LC. Cutaneous sensation of electrical stimulation waveforms. Brain Stimulation. (2021) 14:693–702. doi: 10.1016/j.brs.2021.04.008 33848677 PMC8919780

[21] Minhas P Bansal V Patel J Ho JS Diaz J Datta A. Electrodes for high-definition transcutaneous DC stimulation for applications in drug delivery and electrotherapy, including tDCS. J Neurosci Methods. (2010) 190:188–97. doi: 10.1016/j.jneumeth.2010.05.007 PMC2920288 20488204

[22] Turi Z Ambrus GG Ho K-A Sengupta T Paulus W Antal A. When size matters: large electrodes induce greater stimulation-related cutaneous discomfort than smaller electrodes at equivalent current density. Brain Stimulation. (2014) 7:460–7. doi: 10.1016/j.brs.2014.01.059 24582373

[23] Wu XT Zheng L Jiang L Huang XS Liu YY Xing LH. A dry electrode cap and its application in a steady-state visual evoked potential-based brain-computer interface. Electronics. (2019) 8:1080. doi: 10.3390/electronics8101080

[24] Gao KP Liu JQ Yang HJ Liao LL Jiang CP Zhao N. A novel bristle-shaped semi-dry electrode with low contact impedance and ease of use features for EEG signal measurements. IEEE Trans Biomed Engineering. (2020) 67:750–61. doi: 10.1109/TBME.2019.2920711 31170063

[25] Tallgren P Vanhatalo S Kaila K Voipio J. Evaluation of commercially available electrodes and gels for recording of slow EEG potentials. Clin Neurophysiol. (2005) 116:799–806. doi: 10.1016/j.clinph.2004.10.001 15792889

[26] Guger C Krausz G Allison BZ Edlinger G. Comparison of dry and gel based electrodes for P300 brain-computer interfaces. Front Neurosci. (2012) 6:60. doi: 10.3389/fnins.2012.00060 22586362 PMC3345570

[27] Liu J Lin S Li W Zhao Y Liu D He Z. Ten-hour stable noninvasive brain-computer interface realized by semidry hydrogel-based electrodes. Research. (2022) 2022:9830457. doi: 10.34133/2022/9830457 35356767 PMC8933689

[28] Shen GC Zheng KY Jiang CP Shao SH Zhao N Liu JQ. A gelatin-based hydrogel electrode with high moisturizing ability for wearable EEG recording. IEEE Sensors J. (2023) 23:25689–97. doi: 10.1109/JSEN.2023.3317538

[29] Hu MD Ren J Pan Y Cheng LP Xu X Tan CL. Scaled elastic hydrogel interfaces for brain electrophysiology. Advanced Funct Mater. (2024). doi: 10.1002/adfm.202407926

[30] Khadka N Borges H Zannou AL Jang J Kim B Lee K. Dry tDCS: tolerability of a novel multilayer hydrogel composite non-adhesive electrode for transcranial direct current stimulation. Brain Stimulation. (2018) 11:1044–53. doi: 10.1016/j.brs.2018.07.049 30072144

[31] Valter Y Shahabuddin S McDonald N Roberts B Soussou W Thomas C. Feasibility of Direct Current stimulation through hair using a dry electrode: potential for transcranial Direct Current Stimulation (tDCS) application. IEEE Eng Med Biol Soc Conf Proc. (2021) 43:1584–7. doi: 10.1109/EMBC46164.2021.9630579 34891587

[32] Brunoni AR Nitsche MA Bolognini N Bikson M Wagner T Merabet L. Clinical research with transcranial direct current stimulation (tDCS): challenges and future directions. Brain Stimulation. (2012) 5:175–95. doi: 10.1016/j.brs.2011.03.002 PMC3270156 22037126

[33] Lu HL Zhang YJ Qiu HK Zhang ZL Tan XY Huang P. A new perspective for evaluating the efficacy of tACS and tDCS in improving executive functions: A combined tES and fNIRS study. Hum Brain Mapp. (2024) 45:e26559. doi: 10.1002/hbm.26559 38083976 PMC10789209

[34] Adeel M Chen CC Lin BS Chen HC Liou JC Li YT. Safety of special waveform of transcranial electrical stimulation (TES): *in vivo* assessment. Int J Mol Sci. (2022) 23:6850. doi: 10.3390/ijms23126850 35743291 PMC9224937

[35] Bikson M Grossman P Thomas C Zannou AL Jiang J Adnan T. Safety of transcranial direct current stimulation: evidence based update 2016. Brain Stimulation. (2016) 9:641–61. doi: 10.1016/j.brs.2016.06.004 PMC5007190 27372845

[36] Antal A Alekseichuk I Bikson M Brockmöller J Brunoni AR Chen R. Low intensity transcranial electric stimulation: Safety, ethical, legal regulatory and application guidelines. Clin Neurophysiol. (2017) 128:1774–809. doi: 10.1016/j.clinph.2017.06.001 PMC5985830 28709880

[37] Alonzo A Brassil J Taylor JL Martin D Loo CK. Daily transcranial direct current stimulation (tDCS) leads to greater increases in cortical excitability than second daily transcranial direct current stimulation. Brain Stimulation. (2012) 5:208–13. doi: 10.1016/j.brs.2011.04.006 22037139

[38] Houde F Harvey MP Labrecque PFT Lamarche F Lefebvre A Leonard G. Combining transcranial direct current stimulation and transcutaneous electrical nerve stimulation to relieve persistent pain in a patient suffering from complex regional pain syndrome: a case report. J Pain Res. (2020) 13:467–73. doi: 10.2147/JPR.S226616 PMC7060070 32184651

[39] Pilloni G Vogel-Eyny A Lustberg M Best P Malik M Walton-Masters L. Tolerability and feasibility of at-home remotely supervised transcranial direct current stimulation (RS-tDCS): single-center evidence from 6,779 sessions. Brain Stimulation. (2022) 15:707–16. doi: 10.1016/j.brs.2022.04.014 35470019

[40] Horvath JC Forte JD & Carter O. Evidence that transcranial direct current stimulation (tDCS) generates little-to-no reliable neurophysiologic effect beyond mep amplitude modulation in healthy human subjects: a systematic review. Neuropsychologia. (2015) 66:213–36. doi: 10.1016/j.neuropsychologia.2014.11.021 25448853

[41] Lauro LJR Rosanova M Mattavelli G Convento S Pisoni A Opitz A. tDCS increases cortical excitability: Direct evidence from TMS-EEG. Cortex. (2014) 58:99–111. doi: 10.1016/j.cortex.2014.05.003 24998337

[42] Kuo HI Bikson M Datta A Minhas P Paulus W Kuo MF. Comparing Cortical Plasticity Induced by Conventional and High-Definition 4 x 1 Ring tDCS: A Neurophysiological Study. Brain Stimulation. (2013) 6:644–8. doi: 10.1016/j.brs.2012.09.010 23149292

[43] Paulus W. Transcranial electrical stimulation (tES - tDCS; tRNS, tACS) methods. Neuropsychol Rehabilitation. (2011) 21:602–17. doi: 10.1080/09602011.2011.557292 21819181

[44] Antal A Paulus W. Transcranial alternating current stimulation (tACS). Front Hum Neurosci. (2013) 7:317. doi: 10.3389/fnhum.2013.00317 23825454 PMC3695369

[45] Helfrich RF Knepper H Nolte G Strüber D Rach S Herrmann CS. Selective modulation of interhemispheric functional connectivity by HD-tACS shapes perception. PloS Biol. (2015) 12:e1002031. doi: 10.1371/journal.pbio.1002031 PMC4280108 25549264

[46] Matsumoto H Ugawa Y. Adverse events of tDCS and tACS: A review. Clin Neurophysiol Practice. (2016) 2:19–25. doi: 10.1016/j.cnp.2016.12.003 PMC6123849 30214966

[47] Borckardt JJ Bikson M Frohman H Reeves ST Datta A Bansal V. A pilot study of the tolerability and effects of high-definition transcranial direct current stimulation (HD-tDCS) on pain perception. J Pain. (2012) 13:112–20. doi: 10.1016/j.jpain.2011.07.001 22104190

[48] Zivanovic M Bjekic J Konstantinovic U Filipovic SR. Effects of online parietal transcranial electric stimulation on associative memory: a direct comparison between tDCS, theta tACS, and theta-oscillatory tDCS. Sci Rep. (2022) 12:14091. doi: 10.1038/s41598-022-18376-5 35982223 PMC9388571

[49] Kvasnák E. Perception and Pain Thresholds of tDCS and tACS. Physiol Res. (2019) 68:427–31. doi: 10.33549/physiolres 32118473

[50] Piao Y Ma R Weng YH Fan C Xia XZ Zhang W. Safety evaluation of employing temporal interference transcranial alternating current stimulation in human studies. Brain Sci. (2022) 12:1194. doi: 10.3390/brainsci12091194 36138930 PMC9496688

[51] Baumert A Buchholz N Zinkernagel A Clarke P Schmitt M. Causal underpinnings of working memory and stroop interference control: testing the effects of anodal and cathodal tdcs over the left dlpfc. Cogn Affect Behav Neurosci. (2020) 20:34–8. doi: 10.3758/s13415-019-00726-y PMC7012981 31183619

[52] Schroeter ML Zysset S Kupka T Kruggel F von Cramon DY. Near-infrared spectroscopy can detect brain activity during a color–word matching Stroop task in an event-related design. Hum Brain Mapping. (2002) 17:61–71. doi: 10.1002/hbm.10052 PMC6872032 12203689

[53] Stroop JR. Studies of interference in serial verbal reactions. J Exp Psychol. (1935) 18:643–62. doi: 10.1037/h0054651

[54] Frings C Brinkmann T Friehs MA van Lipzig T. Single session tdcs over the left dlpfc disrupts interference processing. Brain Cognition. (2018) 120:1–7. doi: 10.1016/j.bandc.2017.11.005 29202318

