# Supplementary material for: Evaluation of tolerability and safety of transcranial electrical stimulation with gel particle electrodes in healthy subjects
Source: Front Psychiatry. 2024 Nov 13;15:1441533. doi: 10.3389/fpsyt.2024.1441533 (PMC11599605; doi:10.3389/fpsyt.2024.1441533)
Supplement: Supplementary file 1 [file DataSheet1.pdf]

Table S1. The statistical results of neuropsychological measurements of stimulation duration

| | Measurements (Range or Unit) | Stimulation duration(Mean±SD) 2min | 5min | 7min | 10min | 20min | χ2 | *p* |
|---|---|---|---|---|---|---|---|---|
| TDCS | VAS(0-10) | 4.67±1.91 | 4.47±2.39 | 5.53±2.61 | 4.87±2.67 | 5.00±2.75 | 6.653 | 0.155 |
| | SSR(1-5) | 2.53±0.83 | 2.60±0.83 | 3.00±0.85 | 2.73±0.88 | 2.93±0.88 | 2.813 | 0.590 |
| TACS | VAS(0-10) | 3.47±2.59 | 3.80±2.37 | 4.20±2.40 | 3.67±2.41 | 3.40±1.92 | 4.573 | 0.334 |
| | SSR(1-5) | 2.27±0.7 | 2.53±0.64 | 2.67±0.49 | 2.33±0.62 | 2.27±0.59 | 4.067 | 0.397 |
| | Items of adverse effects (AEs) | | | | | | | |
| TDCS | Headache | 1.67±1.11 | 1.40±0.83 | 1.67±1.18 | 1.67±0.98 | 1.80±1.08 | 2.373 | 0.667 |
| | Neck Pain | 1.00±0.00 | 1.07±0.26 | 1.20±0.77 | 1.07±0.26 | 1.20±0.41 | 0.747 | 0.946 |
| | Scalp Pain | 2.33±1.11 | 2.27±1.10 | 2.73±1.10 | 2.53±1.06 | 2.47±1.19 | 2.880 | 0.578 |
| | Scalp pressure | 1.87±0.92 | 1.80±1.08 | 2.00±1.07 | 1.93±1.10 | 2.07±1.10 | 1.227 | 0.874 |
| | Tingling | 2.73±1.03 | 2.67±0.90 | 3.00±1.07 | 3.13±0.99 | 2.87±0.99 | 3.293 | 0.510 |
| | Burning Sensation | 2.13±1.19 | 2.07±1.10 | 2.07±1.16 | 2.13±1.30 | 2.00±1.13 | 0.280 | 0.991 |
| | Itching Sensation | 1.47±0.92 | 1.40±1.06 | 1.47±0.92 | 1.47±1.06 | 1.40±0.74 | 0.200 | 0.995 |
| | Sleepiness | 1.40±0.74 | 1.53±0.74 | 2.00±0.85 | 1.53±0.74 | 2.20±1.08 | 7.267 | 0.123 |
| | Trouble Concentrating | 1.60±0.91 | 1.47±0.52 | 1.87±0.74 | 1.73±1.10 | 2.13±0.74 | 5.760 | 0.218 |
| | Dizziness | 1.07±0.26 | 1.13±0.35 | 1.20±0.56 | 1.13±0.52 | 1.20±0.41 | 0.467 | 0.977 |
| | Nausea | 1.00±0.00 | 1.13±0.52 | 1.27±0.80 | 1.07±0.26 | 1.27±0.80 | 0.853 | 0.931 |
| | Vibration | 1.07±0.26 | 1.27±0.59 | 1.13±0.35 | 1.13±0.35 | 1.13±0.35 | 0.333 | 0.988 |
| TACS | Headache | 1.47±0.92 | 1.40±0.83 | 1.53±0.99 | 1.47±0.83 | 1.47±0.64 | 0.333 | 0.988 |
| | Neck Pain | 1.00±0.00 | 1.20±0.77 | 1.13±0.35 | 1.07±0.26 | 1.07±0.26 | 0.347 | 0.987 |
| | Scalp Pain | 1.93±0.80 | 2.13±0.99 | 2.20±1.08 | 1.93±0.88 | 1.93±0.88 | 2.040 | 0.728 |
| | Scalp pressure | 2.07±0.88 | 2.20±0.94 | 2.13±0.99 | 2.27±1.10 | 2.07±0.88 | 0.307 | 0.989 |
| | Tingling | 1.87±0.99 | 2.13±1.06 | 2.20±1.01 | 2.00±0.85 | 2.20±0.77 | 2.653 | 0.617 |
| | Burning Sensation | 1.20±0.41 | 1.67±0.90 | 1.67±0.98 | 1.47±0.64 | 1.47±0.92 | 2.973 | 0.562 |
| | Itching Sensation | 1.47±0.99 | 1.40±0.91 | 1.47±0.99 | 1.40±0.83 | 1.33±0.82 | 0.467 | 0.977 |
| | Sleepiness | 1.47±0.74 | 1.87±0.99 | 1.93±0.88 | 1.80±0.77 | 2.27±1.03 | 4.347 | 0.361 |
| | Trouble Concentrating | 1.67±0.82 | 1.87±0.74 | 2.07±0.80 | 2.13±0.92 | 2.20±0.86 | 4.427 | 0.351 |
| | Dizziness | 1.20±0.77 | 1.47±0.92 | 1.40±0.83 | 1.27±0.59 | 1.47±0.74 | 1.427 | 0.840 |
| | Nausea | 1.07±0.26 | 1.07±0.26 | 1.13±0.52 | 1.13±0.52 | 1.07±0.26 | 0.200 | 0.995 |
| | Vibration | 2.67±1.18 | 2.27±1.03 | 2.73±1.16 | 2.60±1.18 | 1.67±1.11 | 8.840 | 0.065 |
| | Items of adverse effects relationship to tES (AEs) | | | | | | | |
| TDCS | Headache | 2.27±1.87 | 1.87±1.55 | 1.80±1.47 | 2.13±1.60 | 2.27±1.62 | 2.427 | 0.658 |
| | Neck Pain | 1.00±0.00 | 1.07±0.26 | 1.27±1.03 | 1.00±0.00 | 1.07±0.26 | 0.213 | 0.995 |
| | Scalp Pain | 3.27±1.75 | 3.53±1.73 | 3.67±1.59 | 3.53±1.73 | 3.93±1.58 | 1.680 | 0.794 |
| | Scalp pressure | 2.80±1.70 | 2.60±1.81 | 2.93±1.79 | 2.87±1.89 | 3.20±1.82 | 0.200 | 0.995 |
| | Tingling | 4.40±1.40 | 4.33±1.40 | 4.33±1.40 | 4.53±1.06 | 4.53±1.13 | 0.640 | 0.959 |
| | Burning Sensation | 2.93±1.83 | 3.00±1.89 | 2.87±1.92 | 2.73±1.87 | 2.60±1.88 | 0.573 | 0.966 |
| | Itching Sensation | 1.80±1.52 | 1.53±1.41 | 2.07±1.58 | 1.67±1.48 | 1.80±1.42 | 1.773 | 0.777 |

| | | | | | | | | |
|---|---|---|---|---|---|---|---|---|
| | Sleepiness | 1.27±0.59 | 1.40±0.83 | 2.07±1.03 | 1.47±0.92 | 2.20±1.42 | 7.200 | 0.126 |
| | Trouble Concentrating | 1.47±0.83 | 1.67±0.90 | 1.87±0.92 | 1.53±0.92 | 2.07±1.10 | 2.333 | 0.675 |
| | Dizziness | 1.20±0.78 | 1.13±0.52 | 1.53±1.41 | 1.07±0.26 | 1.53±1.18 | 1.333 | 0.856 |
| | Nausea | 1.20±0.78 | 1.07±0.26 | 1.47±1.25 | 1.07±0.26 | 1.27±1.03 | 0.573 | 0.966 |
| | Vibration | 1.53±1.41 | 1.80±1.66 | 1.53±1.41 | 1.53±1.41 | 1.47±1.25 | 0.240 | 0.993 |
| TACS | Headache | 1.80±1.42 | 1.73±1.34 | 1.87±1.51 | 1.73±1.22 | 1.80±1.21 | 0.373 | 0.985 |
| | Neck Pain | 1.07±0.26 | 1.27±1.03 | 1.07±0.26 | 1.07±0.26 | 1.20±0.56 | 0.200 | 0.995 |
| | Scalp Pain | 3.47±1.69 | 3.60±1.64 | 3.27±1.75 | 3.60±1.72 | 3.27±1.71 | 0.947 | 0.918 |
| | Scalp pressure | 3.53±1.69 | 3.87±1.60 | 3.67±1.60 | 3.60±1.72 | 3.53±1.73 | 1.107 | 0.893 |
| | Tingling | 3.20±1.90 | 3.60±1.64 | 3.87±1.69 | 3.33±1.76 | 3.93±1.49 | 2.187 | 0.701 |
| | Burning Sensation | 1.80±1.52 | 2.40±1.81 | 2.47±1.92 | 2.20±1.78 | 2.27±1.71 | 2.200 | 0.699 |
| | Itching Sensation | 1.80±1.66 | 1.67±1.45 | 1.87±1.64 | 1.73±1.44 | 1.67±1.40 | 0.653 | 0.957 |
| | Sleepiness | 1.53±0.99 | 2.13±1.19 | 2.07±1.28 | 1.93±1.16 | 2.07±1.34 | 3.253 | 0.516 |
| | Trouble Concentrating | 2.07±1.39 | 2.00±0.93 | 2.33±1.29 | 1.93±1.03 | 2.27±1.34 | 1.000 | 0.910 |
| | Dizziness | 1.40±1.06 | 1.80±1.32 | 1.87±1.55 | 1.33±0.90 | 1.67±1.23 | 1.240 | 0.871 |
| | Nausea | 1.27±0.70 | 1.20±0.78 | 1.20±0.78 | 1.07±0.26 | 1.27±0.70 | 0.307 | 0.989 |
| | Vibration | 3.80±1.78 | 3.47±1.73 | 3.47±1.89 | 3.73±1.79 | 2.20±1.82 | 5.040 | 0.283 |
| | Items of Visual Analog Mood Scale-Revised (VAMS-R) | | | | | | | |
| TDCS | Sorrow | 8.33±22.49 | 10±26.46 | 9.20±24.28 | 5.33±18.07 | 14.00±34.03 | 1.827 | 0.768 |
| | Bewilderment | 9.33±23.44 | 5.67±21.95 | 8.00±21.48 | 5.40±18.05 | 8.33±21.35 | 0.360 | 0.986 |
| | Fear | 12.33±26.65 | 8.00±21.45 | 5.33±20.66 | 5.67±16.78 | 5.33±20.66 | 0.787 | 0.940 |
| | Happy | 39.00±32.96 | 27.33±28.40 | 35.67±33.85 | 34.33±28.84 | 29.33±31.73 | 1.560 | 0.816 |
| | Fatigued | 20.33±28.31 | 28.67±32.04 | 44.20±33.24 | 47.67±30.41 | 54.00±31.35 | **13.667** | **0.008** |
| | Anger | 6.67±17.59 | 1.33±5.16 | 6.67±25.82 | 4.67±12.46 | 5.33±13.56 | 0.533 | 0.970 |
| | Strain | 14.00±23.54 | 6.33±19.32 | 6.40±18.83 | 9.20±24.52 | 9.00±22.38 | 1.640 | 0.802 |
| | Full of energy | 45.93±33.83 | 33.00±35.04 | 29.67±32.98 | 32.2±28.17 | 28.33±27.04 | 4.720 | 0.317 |
| TACS | Sorrow | 4.67±18.07 | 10.67±27.89 | 5.33±20.66 | 6.87±21.85 | 11.67±23.58 | 2.707 | 0.608 |
| | Bewilderment | 4.67±18.07 | 9.33±25.76 | 5.33±20.66 | 12.13±25.76 | 4.33±15.45 | 1.693 | 0.792 |
| | Fear | 5.67±20.60 | 8.67±23.56 | 5.33±20.66 | 8.40±22.20 | 4.00±15.49 | 0.413 | 0.981 |
| | Happy | 42.67±32.83 | 24.67±28.25 | 31.33±33.57 | 34±26.34 | 29.67±29.79 | 3.187 | 0.527 |
| | Fatigued | 21.40±23.92 | 33.67±38.75 | 38.4±30.73 | 38.93±34.40 | 55.00±39.60 | 8.413 | 0.078 |
| | Anger | 2.00±7.75 | 3.33±12.91 | 6.67±25.82 | 3.33±12.91 | 0.00±0.00 | 0.187 | 0.996 |
| | Strain | 5.67±19.35 | 8.67±23.56 | 8.00±21.45 | 11.40±29.51 | 4.67±18.07 | 0.453 | 0.978 |
| | Full of energy | 51.53±33.66 | 32.00±37.41 | 25.33±28.00 | 33.00±28.59 | 22.00±24.55 | **9.906** | **0.042** |
| | Stroop task | | | | | | | |
| TDCS | Reaction time | 1.01±0.34 | 0.95±0.22 | 0.93±0.23 | 0.95±0.19 | 0.95±0.19 | 0.907 | 0.924 |
| | Percent of Correct | 0.97±0.03 | 0.97±0.02 | 0.97±0.02 | 0.97±0.03 | 0.97±0.01 | 2.933 | 0.569 |
| TACS | Reaction time | 0.99±0.33 | 0.97±0.26 | 0.94±0.18 | 0.94±0.20 | 0.96±0.24 | 0.867 | 0.929 |
| | Percent of Correct | 0.97±0.03 | 0.97±0.02 | 0.97±0.02 | 0.97±0.02 | 0.97±0.01 | 2.293 | 0.682 |

Table S2. The statistical results of neuropsychological measurements of stimulation types

| **Measurements (Range or Unit)** | Before (mean ± SD) | After (mean ± SD) | *Z* | *P* |
|---|---|---|---|---|
| MoCA(0-30) | 28.73±0.96 | 28.73±0.96 | 0 | 1 |
| SAS(1-4) | 39.13±4.03 | 39±5.52 | 0.229 | 0.814 |
| | TDCS (mean ± SD) | TACS (mean ± SD) | *Z* | *P* |
| VAS(0-10) | 4.91±2.47 | 3.71±2.34 | **2.867** | **0.001** |
| SSR(1-5) | 2.76±0.86 | 2.41±0.61 | **2.048** | **0.033** |
| Items of adverse effects (AEs) | | | | |
| Headache | 1.64±1.02 | 1.47±0.83 | **2.780** | **0.006** |
| Neck Pain | 1.11±0.42 | 1.09±0.41 | 0.138 | 1.000 |
| Scalp Pain | 2.47±1.09 | 2.03±0.91 | **1.099** | **<0.001** |
| Scalp pressure | 1.93±1.03 | 2.15±0.94 | **-2.439** | **0.018** |
| Tingling | 2.88±0.99 | 2.08±0.93 | **5.526** | **<0.001** |
| Burning Sensation | 2.08±1.15 | 1.49±0.79 | **4.399** | **<0.001** |
| Itching Sensation | 1.44±0.92 | 1.41±0.89 | 0.332 | 0.838 |
| Sleepiness | 1.73±0.88 | 1.87±0.91 | -1.326 | 0.205 |
| Trouble Concentrating | 1.76±0.84 | 1.99±0.83 | **-2.215** | **0.029** |
| Dizziness | 1.15±0.43 | 1.36±0.77 | **-2.863** | **0.003** |
| Nausea | 1.15±0.56 | 1.09±0.37 | 1.175 | 0.359 |
| Vibration | 1.15±0.39 | 2.39±1.17 | **-5.976** | **<0.001** |
| Items of adverse effects relationship to tES (AEs) | | | | |
| Headache | 2.07±1.60 | 1.79±1.31 | **2.492** | **0.012** |
| Neck Pain | 1.08±0.49 | 1.13±0.55 | -0.776 | 0.469 |
| Scalp Pain | 3.59±1.64 | 3.44±1.66 | 1.218 | 0.229 |
| Scalp pressure | 2.88±1.76 | 3.64±1.62 | **-3.323** | **<0.001** |
| Tingling | 4.43±1.25 | 3.59±1.68 | **4.495** | **<0.001** |
| Burning Sensation | 2.83±1.83 | 2.23±1.72 | **3.259** | **<0.001** |
| Itching Sensation | 1.77±1.45 | 1.75±1.48 | 0.127 | 0.905 |
| Sleepiness | 1.68±1.04 | 1.95±1.18 | -1.750 | 0.076 |
| Trouble Concentrating | 1.72±0.94 | 2.12±1.19 | **-2.455** | **0.013** |
| Dizziness | 1.29±0.93 | 1.61±1.22 | **-2.836** | **0.003** |
| Nausea | 1.21±0.81 | 1.20±0.66 | 0.000 | 1.000 |
| Vibration | 1.57±1.40 | 3.33±1.85 | **-5.569** | **<0.001** |
| Items of Visual Analog Mood Scale-Revised (VAMS-R) | | | | |
| Sorrow | 9.37±25.07 | 7.84±22.21 | 0.473 | 0.636 |
| Bewilderment | 7.35±20.80 | 7.16±21.17 | 0.198 | 0.844 |
| Fear | 7.33±21.06 | 6.41±20.20 | 1.071 | 0.285 |
| Happy | 33.13±30.68 | 32.47±30.05 | 0.608 | 0.545 |

| | | | | |
|---|---|---|---|---|
| Fatigued | 38. 97±32. 78 | 37. 48±34. 78 | 0. 561 | 0. 577 |
| Anger | 4. 93±16. 06 | 3. 07±14. 33 | 1. 260 | 0. 227 |
| Strain | 8. 99±21. 42 | 7. 68±22. 25 | 0. 789 | 0. 437 |
| Full of energy | 33. 83±31. 35 | 32. 77±31. 66 | 0. 817 | 0. 417 |
| Stroop task | | | | |
| Reaction time | 0. 96±0. 22 | 0. 96±0. 21 | −0. 312 | 0. 762 |
| Percent of Correct | 0. 97±0. 02 | 0. 97±0. 02 | −1. 164 | 0. 252 |

**Supplementary Experiment:**

The purpose of using the MoCA scale in this experiment is to detect whether entire participation in the experiment results in mild cognitive impairment in the participants. Since all participants in this study had more than 15 years of education, a score of 26 was used as the cutoff diagnostic value (1, 2). Although all MoCA results exceeded 26 points, not all scores were perfect. Please refer to the MoCA score distribution chart below.

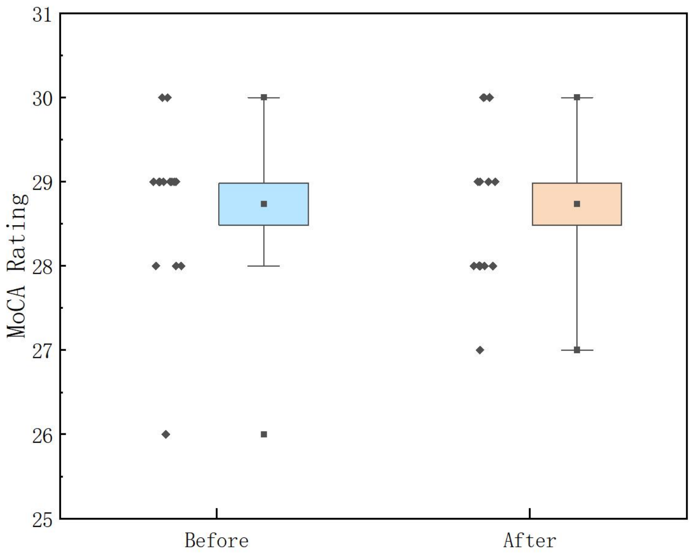


Figure S1. Comparison of the distribution of MoCA scores before and after the entire tES experiment.

In this study, the Stroop task was used as the online task during stimulation, as it is a task sensitive to cognitive changes (3). The comparison of the percent of correct for the first 200 trials and the last 200 trials throughout the experimental phase showed no significant change ($Z = -1.84588$, $p = 0.06372$), which can support the MoCA results ($Z = 0$, $p = 1$). Finally, follow-up visits with participants one week and one month after the experiment revealed no AEs.

In the supplementary experiment, selecting 5 minutes and 10 minutes durations for pure tES. Participants did not perform the Stroop task while receiving electrical stimulation. Cognitive changes were assessed using MoCA, Stroop, Digit Symbol Substitution Test (DSST), and Trail Making Test Part B (TMT-B) (3-6) before and after the entire stimulation phase. A total of 15 participants, and they were required to

remain awake during the experiment. One participant's data was excluded due to falling asleep during the session. All other experimental conditions and statistical analysis methods were consistent with those in the original manuscript.

The supplementary experiment results showed that MoCA ($Z$ = -1.81956, p = 0.07813), Stroop ($Z$ = -1.69664, p = 0.08545), DSST ($Z$ = 0.70122, p = 0.48096), and TMT-B ($Z$ = 1.69497, p = 0.09058) all demonstrated no significant differences in cognitive assessments.

The experimental design in this study was that the stimulation duration was the same as the session (and Stroop) duration. Participants underwent sessions of different durations when receiving stimulation for varying lengths of time. In this context, fatigue effect was observed. The purpose is to help participants focus their attention on the task during electrical stimulation, preventing excessive tension from amplifying the experimental experience. The Stroop results also indicate that participants were able to concentrate on the task, balancing their focus of attention to ensure the comparability and reliability of the experimental results.

Admittedly, as the reviewer mentioned, the Stroop task during the experimental process, aside from the tES, may also affect fatigue, and this factor is indeed possible. Therefore, we conducted a supplementary comparison experiment, selecting 5 minutes and 10 minutes durations for pure tES. Participants did not perform the Stroop task while receiving electrical stimulation. A total of 15 participants, and they were required to remain awake during the experiment. One participant's data was excluded due to falling asleep during the session. All other experimental conditions and statistical analysis methods were consistent with those in the original manuscript. The results showed a significant difference in the fatigue item measure when tACS was applied ($Z$ = -1.93173, p = 0.0459), while no significant differences were observed in other measures, indicating that electrical stimulation has a significant impact on fatigue item.

Thus, although the Stroop task may have influenced the results, we believe that the effect of electrical stimulation on fatigue remains evident.

References

1. Carson N, Leach L, Murphy KJ. A re-examination of Montreal Cognitive Assessment (MoCA) cutoff scores. Int J Geriatric Psychiatry. (2018) 33:379-88. doi: 10.1002/gps.v33.2.

2. Nasreddine ZS, Phillips NA, Bedirian V, Charbonneau S, Whitehead V, Collin I, et al. The montreal cognitive assessment, moCA: A brief screening tool for mild cognitive impairment. J Am Geriatrics Society. (2019) 67:1991-1. doi: 10.1111/jgs.15925.

3. Osimani A, Alon A, Berger A, Abarbanel JM. Use of the Stroop phenomenon as a diagnostic tool for Malingering. J Neurol Neurosurg Psychiatry. (1997) 62:617-21. doi: 10.1136/jnnp.62.6.617.

4. Daderwal MC, Sreeraj VS, Suhas S, Rao NP, Venkatasubramanian G. Montreal Cognitive Assessment (MoCA) and Digit Symbol Substitution Test (DSST) as a

screening tool for evaluation of cognitive deficits in schizophrenia. Psychiatry Res. (2022) 316. doi: 10.1016/j.psychres.2022.114731.

5. Giovagnoli AR, DelPesce M, Mascheroni S, Simoncelli M, Laiacona M, Capitani E. Trail making test: Normative values from 287 normal adult controls. Ital J Neurological Sci. (1996) 17:305-9. doi: 10.1007/BF01997792.

6. Jaeger J. Digit symbol substitution test: the case for sensitivity over specificity in neuropsychological testing. J Clin Psychopharmacol. (2018) 38:513-9. doi: 10.1097/JCP.0000000000000941.
